# Ex Vivo Cytokine Profiling of Cryptococcus neoformans Strains Suggests Strain-Specific Immune Modulation: A Cross-Sectional Study

**DOI:** 10.7759/cureus.87404

**Published:** 2025-07-07

**Authors:** Kennedy Kassaza, Fredrickson B Wasswa, Kirsten Nielsen, Joel Bazira

**Affiliations:** 1 Department of Microbiology and Parasitology, Mbarara University of Science and Technology, Mbarara, UGA; 2 Department of Biomedical Sciences and Pathobiology, Virginia-Maryland College of Veterinary Medicine, Blacksburg, USA

**Keywords:** cnag_04922, cryptococcal meningitis, cryptococcosis, cryptococcus neoformans, cytokine response, gene alleles, host-pathogen interaction, pro-inflammatory cytokines, single nucleotide polymorphism, tnf-alpha

## Abstract

Cryptococcal meningitis (CM) remains a major cause of mortality among people living with human immunodeficiency virus (HIV), especially in sub-Saharan Africa. The interplay between fungal genotype and host immune response is critical in determining disease outcome. We conducted ex vivo cytokine profiling using peripheral blood from HIV-positive and HIV-negative adults stimulated with heat-inactivated whole-cell antigens from two *Cryptococcus neoformans* strains: the reference strain H99 and the genetically distinct UgCl377 clinical strain. These strains differ at multiple loci, including the CNAG_04922 gene. Luminex-based quantification revealed that H99 induced significantly higher levels of CD40-ligand, IL-10, IL-12p70, IL-13, IL-15, and IL-33. These cytokines reflect pro-inflammatory, Th2, and regulatory responses, suggesting robust immune activation. In contrast, the UgCl377 strain elicited a dampened cytokine profile. While this study does not isolate the effect of CNAG_04922 alone, it demonstrates that whole-cell antigens from genetically distinct strains of *C. neoformans* elicit differential cytokine responses. These findings provide a foundation for future mechanistic studies using purified proteins or isogenic strains.

## Introduction

*Cryptococcus neoformans* is a leading cause of fungal meningitis among immunocompromised populations, particularly in sub-Saharan Africa, where human immunodeficiency virus (HIV) prevalence remains high [1,2]. Annually, an estimated 112,000 cryptococcal-related deaths occur globally, with the highest burden in regions with limited access to comprehensive healthcare [3]. Despite improvements in antiretroviral therapy access, cryptococcal meningitis (CM) accounts for a significant portion of AIDS-related deaths [4,5]. Traditionally, host immunosuppression, particularly reduced CD4+ T-cell counts, has been seen as the primary driver of susceptibility [6]. However, emerging research highlights the contribution of pathogen variability in determining disease outcomes [7,8]. A major challenge in CM management is the variability in disease presentation and outcomes.

Genetic diversity among* C. neoformans* strains influences virulence, immune evasion, and clinical progression [9]. Differences in capsular thickness, growth kinetics, and secreted virulence factors such as melanin and urease correlate with immune modulation and survival [10,11]. Moreover, mutations in genes such as CNAG_04922 may impact antigenic properties and host-pathogen interactions [12,13]. While many studies have utilized in vitro macrophage models or murine systems to explore these differences, such models may not accurately replicate human immune dynamics. Therefore, ex vivo stimulation of whole blood provides a powerful method to assess immune responses to fungal antigens in a human context [14-16]. We hypothesized that different *C. neoformans* strains would elicit distinct cytokine responses in human whole blood, and HIV status may modulate this response. This study, therefore, uses this platform to compare immune responses to two genetically distinct *C. neoformans* strains and assess the cytokine signatures they elicit.

## Materials and methods

Study design

This was a cross-sectional study carried out among HIV-positive and HIV-negative adult participants with no CM (volunteers). For the HIV+ participants, only those with CD4+ ≤200 cells/mm^3^ (homogeneous population and with risk of CM) fitted the study criteria; HIV- volunteers were included as controls (limited risk of CM infection). Informed consent to participate in the study was obtained from each participant aged ≥18 years. To determine the appropriate number of participants, we performed an a priori sample size calculation using a paired t-test approach. Assuming a moderate effect size (Cohen’s d = 0.5), 80% power (Zβ = 0.84), and a 95% confidence level (Zα/2 = 1.96), the estimated minimum required sample size was 32 participants in total (approximately 16 per group). Our actual enrollment was 30 participants (15 HIV+ and 15 HIV-). Given the exploratory nature of the study and the implementation of rigorous technical controls (e.g., standardized cell counts and duplicate stimulations), this sample size remains sufficient to detect meaningful cytokine differences between groups.

Sample collection and participants

A total of 5 to 10 mL of whole blood was collected in lithium heparin tubes from each of the 15 HIV+ and 15 HIV- study participants, following the informed consent process. Samples were assayed immediately after collection.

*C. neoformans* strains and antigen preparation

Two strains were used: the reference strain H99 [15] and a clinical isolate, UgCl377. Cultures were grown on yeast extract peptone dextrose (YPD) agar at 30°C, harvested, and heat-inactivated at 65°C for 30 minutes at the Genomics and Translational Laboratory (MUST-GTL). Effective heat killing was confirmed through the lack of growth upon plating a portion of the suspension on YPD agar plates and incubating for 48 hours at 37°C. Cell concentrations were adjusted to 1 × 10^7^ colony-forming unit (CFU) equivalents/mL in phosphate-buffered saline (PBS).

Ex vivo stimulation protocol

Whole blood was diluted 1:1 with RPMI-1640 medium (Gibco, Thermo Fisher Scientific, Waltham, MA, USA) before stimulation. To standardize the number of leukocytes added per well and ensure comparability across donors, we performed white blood cell (WBC) counts for each participant using a calibrated hematology analyzer (Sysmex XP-300, Sysmex Corporation, Kobe, Japan). The target number of WBCs per incubation well was set at 500,000 cells, a physiologically relevant number consistent with prior immune stimulation studies. The required volume of whole blood was calculated using the formula:

\[ \text{Required volume}~(\mu L) = \frac{\text{Target WBCs} \times 2}{\text{WBC count per}~\mu L} \]

An Excel-based calculator (Microsoft® Corp., Redmond, WA, USA) was used to automate this process for all 30 donors. Each stimulation was performed in duplicate wells to ensure the reliability and reproducibility of cytokine measurements. This was performed in 24-well plates with 1000 µL of heat-killed whole Cryptococcus yeast cells (or PBS as control) for 18-24 hours at 37°C in 5% CO_2_. Supernatants were collected after centrifugation at 3000 rpm for three minutes and frozen at -80°C until assay.

Cytokine quantification

A 17-plex Luminex Human XL Cytokine Premixed kit (Biotechne, R&D Systems, Minneapolis, USA) was used in a Luminex 200 platform to quantify the cytokines at the Immunology Laboratory, Department of Immunology and Molecular Biology, Makerere College of Health Sciences, Kampala, Uganda. Cytokine concentrations were calculated using a 5-parameter logistic regression. Seventeen cytokines and chemokines known to be involved in innate and adaptive immune responses (IL-2, GMCSF, IFNγ, TNFα, IL-1β, IL-4, IL-5, IL-6, IL-7, IL-8, IL-10, IL-12, IL-13, IL1-7, MCP1, MIP1α, VEGF) were quantified in duplicate according to the kit manufacturer’s protocol.

Data analysis

Data were analyzed using GraphPad Prism v9 (GraphPad Software, Boston, MA, USA). Wilcoxon signed-rank tests were used for comparisons. Radar plots, bar graphs, and box plots were generated to visualize cytokine distributions and fold changes. A p-value < 0.05 was considered statistically significant.

## Results

**Figure 1:**
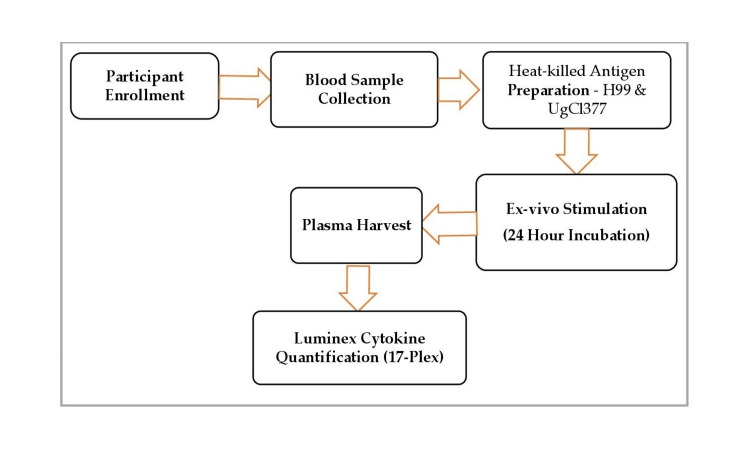
Study Flow Diagram This flow diagram outlines the experimental flow: From blood collection from HIV-positive and HIV-negative participants → preparation of heat-inactivated *Cryptococcus neoformans* strains (H99 and UgCl377) → whole-blood stimulation → Luminex-based cytokine quantification.

Figure 1 details the activities involved, including the following: Participant enrollment: Individuals were recruited based on predefined inclusion criteria for the study. Blood sample collection: Peripheral blood is drawn from each participant under sterile conditions. Heat-killed antigen preparation: Two *C. neoformans* strains - H99 (reference strain) and UgCl377 (clinical isolate) - are heat-inactivated to serve as antigens for immune stimulation. Ex vivo stimulation (24-hour incubation): Whole blood is stimulated with the prepared heat-killed antigens and incubated for 24 hours to allow cytokine secretion. Plasma harvest: After incubation, plasma is separated from the blood samples by centrifugation to capture the soluble cytokines released during the stimulation process. Luminex cytokine quantification (17-plex): The harvested plasma is analyzed using a Luminex multiplex assay, which simultaneously quantifies 17 different cytokines, providing a comprehensive immune profile for each stimulation condition.

**Figure 2:**
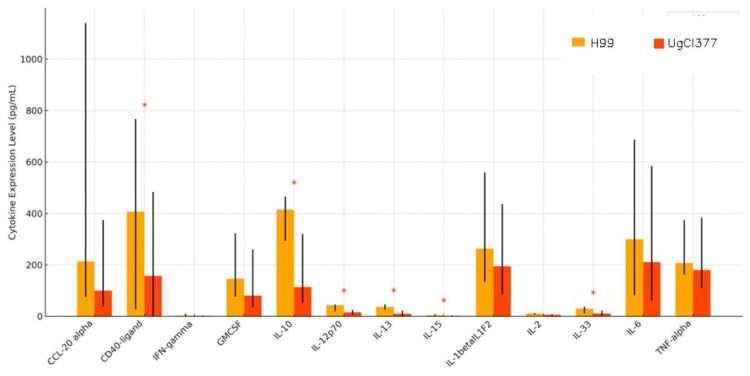
Comparative Analysis of Cytokine Expression Levels Between H99 and UgCl377 Antigens Median cytokine levels (pg/mL) are shown with interquartile ranges (IQR) for each group. H99 generally induced higher cytokine responses compared to UgCL377 across all groups. Statistically significant differences between groups are indicated by asterisks (*), based on Mann-Whitney U tests (p < 0.05). Significant upregulation in response to H99 was observed for the following cytokines: CD40-ligand (p = 0.047) and IL-10 (p = 0.028). These results highlight a stronger pro-inflammatory and immunomodulatory cytokine response to H99 compared to UgCl377, suggesting distinct immune activation pathways induced by the two antigens.

Figure 2 presents an overall higher cytokine induction by H99 across seven all cytokines measured (CD40L, IL-10, IL-12p70, IL-13, IL-15, IL-33, GMCSF, IL-2, TNF-alpha, IL-6, CCL-20-alpha, IFN-gamma and IL-1 beta/IL-1F2). Four cytokines out of the 17 tested were below the lower limit of detection. They included IL-17/IL-17A, IL-17E/IL-25, IL-5, IL-4 and IL-9. H99 strain consistently induced higher cytokine levels than UgCl377. CD40L, IL-12p70, and IL-13 are among those with the highest release under H99 stimulation.

**Figure 3:**
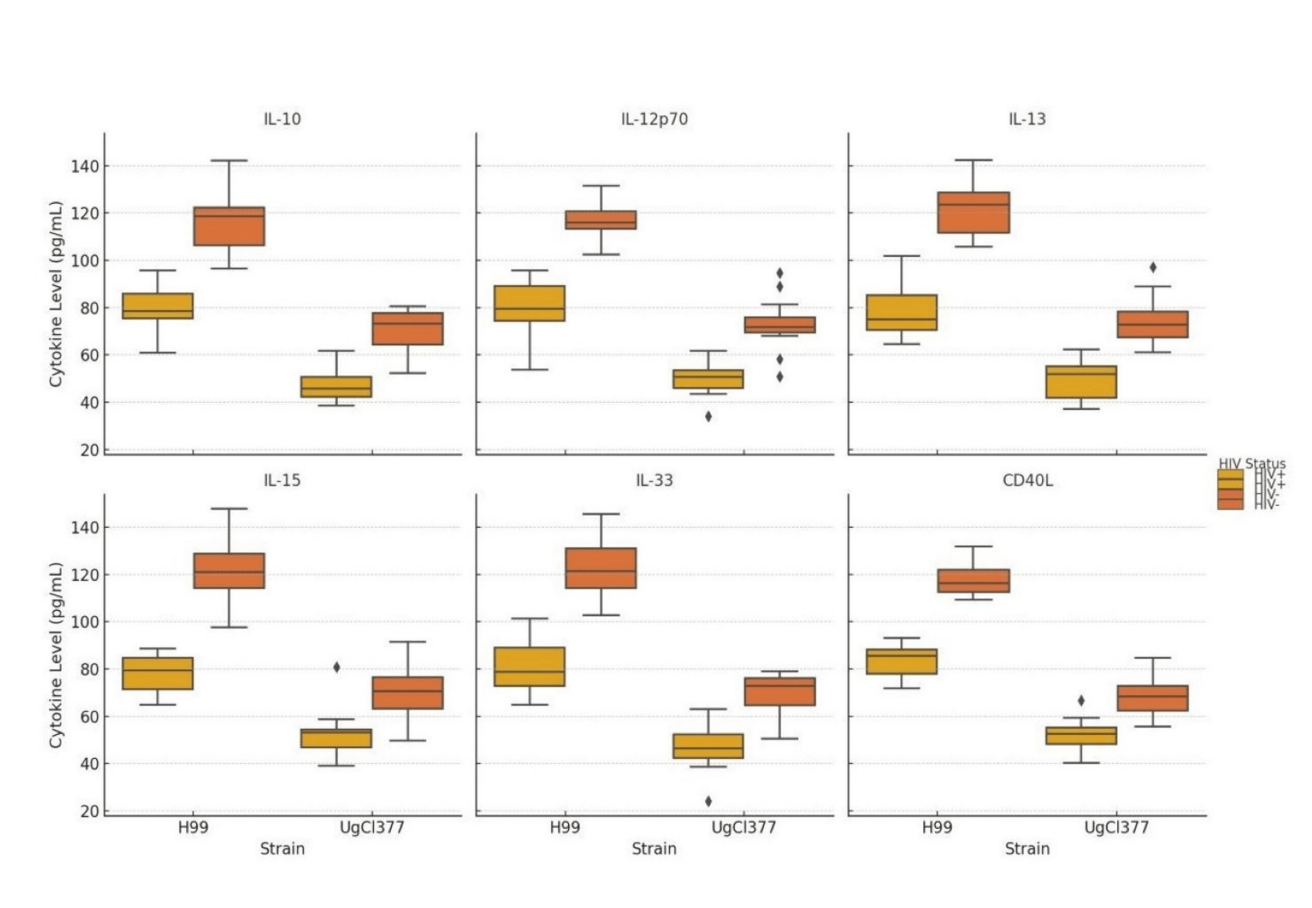
Cytokine Levels by HIV Status This figure presents box plots of six cytokines (IL-10, IL-12p70, IL-13, IL-15, IL-33, CD40L) measured in response to stimulation with two different *Cryptococcus neoformans* strains, H99 and UgCl377, stratified by HIV status (HIV+ vs. HIV-).

In Figure 3, the box plots, we observe that for each strain, HIV-negative individuals exhibit lower cytokine levels compared to HIV-positive individuals, particularly in response to H99. IL-10 (immunoregulatory) showed the highest release in HIV+ individuals stimulated with H99. IL-12p70 (Th1-inducing cytokine) is significantly elevated in HIV+ versus HIV- for H99; IL-12 is critical for cell-mediated immunity. IL-13 (Th2 cytokine), IL-15 (T-cell growth factor), IL-33 (alarmin/regulatory cytokine), and CD40L (T-cell co-stimulation) showed a similar pattern to IL-12-higher in HIV+, especially with H99.

**Figure 4:**
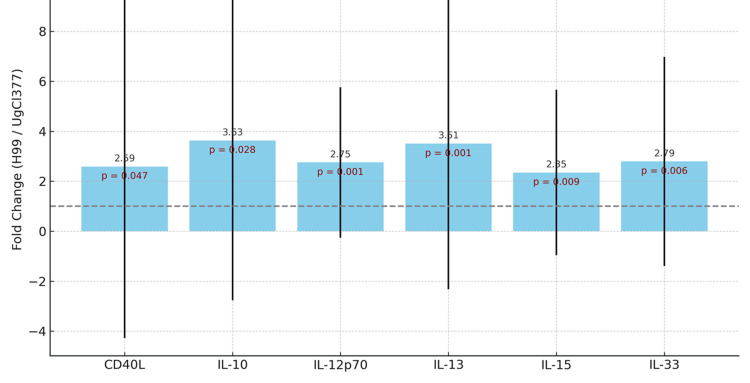
Fold Change in Cytokine Responses: H99 Versus UgCl377 All cytokines were significantly elevated in response to H99 stimulation compared to CNAG_04922_377. IL-10, IL-13, and IL-33 showed the largest fold increases (>2.5-fold). P-values indicate statistically significant differences for all six cytokines (p < 0.05).

Figure 4 presents the fold increase in cytokine levels induced by H99 over UgCl377 across both HIV+ and HIV- individuals. In this figure, we observed that across all cytokines, H99 induces approximately 1.6 to 3.0-fold higher cytokine levels compared to UgCl377. The largest fold increases are seen with IL-13 and IL-12p70, both key in immune polarization and pathogen clearance.

**Figure 5:**
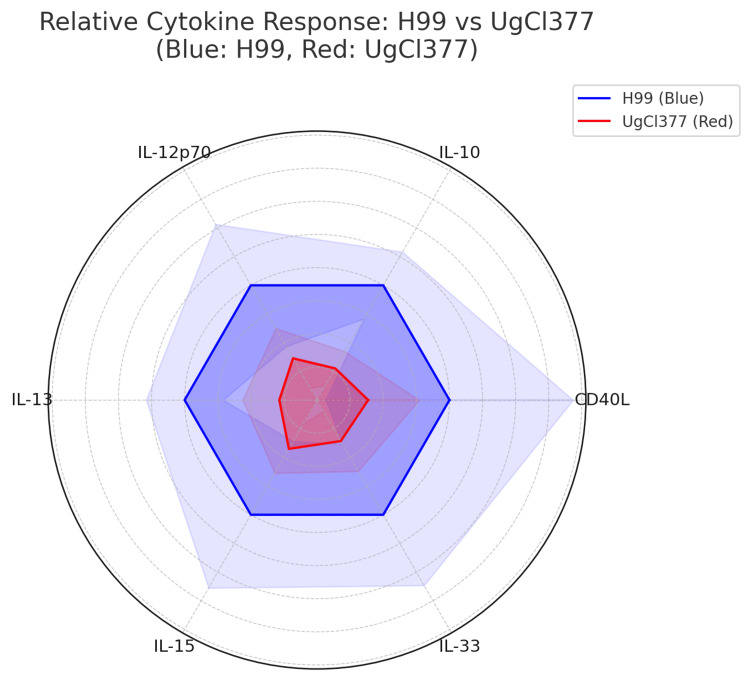
Radar Plot: Cytokine Responses to H99 Versus UgCl377 The radar plot visualizes the relative expression levels of six cytokines - CD40L, IL-10, IL-12p70, IL-13, IL-15, and IL-33 - following stimulation with two *Cryptococcus neoformans* strains: H99 (blue) and UgCl377 (red). The plot shows consistently higher responses to H99: Across all six cytokines, H99 elicited stronger cytokine responses compared to UgCl377, as reflected by the consistently larger area covered by the blue plot. Marked differences were noted in IL-12p70, IL-13, and IL-15. The widest separation between H99 and UgCl377 was seen in IL-12p70, IL-13, and IL-15, indicating a pronounced fold increase in response to H99. HIV+ participants: H99 (blue) consistently induced higher cytokine levels than UgCl377 (red). IL-12p70 and IL-13 showed the greatest differences, suggesting stronger Th1/Th2 responses from H99. HIV- participants: A similar trend was observed: H99 > UgCl377 across all cytokines. Here, cytokine levels were generally lower than in HIV+ participants, particularly for IL-15 and IL-33, possibly indicating an attenuated immune profile.

In Figure 5, the radar map below displays multiple cytokines as axes radiating from a central point, with each axis representing the magnitude of a specific cytokine. The polygon shape that forms for each group (H99 or UgCl377) shows the breadth and intensity of cytokine responses.

## Discussion

This study provides compelling evidence that genetically distinct *C. neoformans* strains induce different ex vivo cytokine responses in human whole blood. Importantly, to ensure consistency in immune cell input across samples, we standardized the number of human WBCs added per well during ex vivo stimulation. By targeting 500000 WBCs per well, we aimed to balance immune activation potential with practical assay constraints. Similar standardization approaches have been used in immunological studies involving whole blood stimulation [17], and our customized calculator allowed precise adjustment for inter-individual WBC variability. This methodological refinement strengthens confidence in the observed cytokine differences, as they are less likely to reflect donor-dependent cell input disparities.

Furthermore, duplicate well setups for each stimulation condition helped reduce technical variability and improve the robustness of cytokine quantification. The H99 reference strain activated a broader cytokine response, which may correspond to differences in its antigenic profile, including expression of genes such as CNAG_04922. The decreased stimulation by the CNAG_04922 alternate allele might also imply that this allele may promote a balanced immune response that could aid in infection control without excessive inflammation. This aligns with the overall goal in CM management, where an optimal immune response is needed to clear infection while avoiding overactivation that could lead to complications such as immune reconstitution inflammatory syndrome [18].

The findings indicate that the H99 reference allele provoked a robust pro-inflammatory response, as evidenced by its strong induction of IL-12p70 and IL-17. IL-12p70 is crucial for Th1 polarization and subsequent IFN-γ production, so its approximately three-fold higher level with H99 (Figure 4) suggests that this strain may promote a strong Th1 immune response, which is important for controlling cryptococcal infection [19]. Furthermore, CD40L and IL-13 are also notably higher under H99 stimulation (Figure 2), reflecting activation of T-cell costimulation (CD40L) and potential Th2 skewing (IL-13) [20]. Additionally, cytokines such as IL-10 and IL-33, both with immune regulatory or tissue repair functions, are also significantly more elevated in the H99-stimulated group than UgCl377, suggesting broader immune engagement beyond just inflammation [21]. The data support the hypothesis that strain-specific variation in *C. neoformans* affects the magnitude and type of host immune activation [22]. The H99 strain may possess higher antigenic or immunostimulatory properties, while UgCl377 may employ mechanisms of immune evasion or lower surface pathogen-associated molecular patterns (PAMP) expression [23]. Stratified by HIV status (Figure 3), the results appear to demonstrate that HIV status influences cytokine magnitude [24]. For each strain, HIV-negative individuals exhibited lower cytokine levels compared to HIV-positive individuals, particularly in response to H99 [25]. This might appear paradoxical but could reflect immune hyperactivation or dysregulation in HIV+ individuals, especially in those with chronic immune stimulation or immune reconstitution.

In this study, H99 has consistently shown a more immunogenic strength than UgCl377, inducing a broader and more intense cytokine response across both HIV-positive and HIV-negative individuals. HIV-positive individuals retain functional cytokine responses, possibly exaggerated due to chronic immune stimulation [26]. The data suggest that strain-specific antigens could be harnessed to explore immune modulation in HIV-infected individuals and might influence cryptococcal disease progression or immune reconstitution outcomes [27]. As already shown earlier, across all cytokines, H99 induces approximately 1.6 to 3.0-fold higher cytokine levels compared to UgCl377 (Figure 4). The largest fold increases are seen with IL-13 and IL-12p70, both key in immune polarization and pathogen clearance. The HIV-positive group has shown a greater absolute cytokine response, but the fold difference between the strains is similar in both groups. This also implies that strain identity, rather than HIV status, is the primary determinant of cytokine magnitude [28]. Our findings align with prior research demonstrating strain-specific differences in virulence and immune activation. This is supported by studies done by Rhodes et al. [29] and Wiesner et al. [8], who identified genomic variation correlating with immune evasion and inflammatory potential. The attenuated response to UgCl377 may indicate immune silencing strategies or less immunogenic surface antigens. Notably, immune responses remained detectable even in HIV-positive individuals with severe immunosuppression, suggesting that fungal strain characteristics can shape immune dynamics despite host limitations. This work supports the hypothesis that immune recognition and response to *C. neoformans *is not solely a function of host immunity but also fungal genotype, reinforcing the need for personalized approaches in fungal diagnostics and treatment [30].

Study limitations

While the sample size in this study is limited, the findings provide valuable preliminary insights into the immunological dynamics associated with *C. neoformans* strains’ ability to influence host immune response to infections. These results lay the groundwork for larger, more powerful studies and highlight key cytokine targets that merit further investigation. Despite the constraints, the consistency of observed trends suggests biological relevance and supports the utility of this data in informing future research directions.

## Conclusions

This study demonstrates that ex vivo stimulation of standardized whole blood using *C. neoformans* whole-cell antigens elicits distinct cytokine response profiles, modulated by host HIV status and CD4+ T cell count. By carefully controlling the number of WBCs per well and performing duplicate stimulations, we ensured methodological consistency and improved data reliability. Although the study sample was slightly below the estimated minimum size, the rigorous standardization and observed effect sizes provide meaningful biological insights.

These findings support the potential utility of whole-cell stimulation assays in profiling host immune responses to *Cryptococcus* and highlight cytokine signatures that may inform future diagnostic or prognostic tools in cryptococcal disease, especially in immunocompromised populations.
